# Developing a Method to Estimate the Downstream Metabolite Signals from Hyperpolarized [1-^13^C]Pyruvate

**DOI:** 10.3390/s22155480

**Published:** 2022-07-22

**Authors:** Ching-Yi Hsieh, Cheng-Hsuan Sung, Yi-Liang (Eric) Shen, Ying-Chieh Lai, Kuan-Ying Lu, Gigin Lin

**Affiliations:** 1 Medical Imaging Research Center, Institute for Radiological Research, Chang Gung University, Taoyuan 333, Taiwan; chsieh2016@mail.cgu.edu.tw (C.-Y.H.); a0927001062@gmail.com (C.-H.S.); 2 Clinical Metabolomics Core Laboratory, Chang Gung Memorial Hospital at Linkou, Taoyuan 333, Taiwan; fantacy52317@gmail.com; 3 Department of Radiation Oncology and Proton Therapy Center, Chang Gung Memorial Hospital at Linkou, Chang Gung University, Taoyuan 333, Taiwan; patience.tw@gmail.com; 4 Department of Medical Imaging and Intervention, Chang Gung Memorial Hospital at Linkou, Chang Gung University, Taoyuan 333, Taiwan; cappolya@gmail.com

**Keywords:** hyperpolarized carbon-13, metabolites, apparent exchange rate, kinetic model

## Abstract

Hyperpolarized carbon-13 MRI has the advantage of allowing the study of glycolytic flow in vivo or in vitro dynamically in real-time. The apparent exchange rate constant of a metabolite dynamic signal reflects the metabolite changes of a disease. Downstream metabolites can have a low signal-to-noise ratio (SNR), causing apparent exchange rate constant inconsistencies. Thus, we developed a method that estimates a more accurate metabolite signal. This method utilizes a kinetic model and background noise to estimate metabolite signals. Simulations and in vitro studies with photon-irradiated and control groups were used to evaluate the procedure. Simulated and in vitro exchange rate constants estimated using our method were compared with the raw signal values. In vitro data were also compared to the Area-Under-Curve (AUC) of the cell medium in ^13^C Nuclear Magnetic Resonance (NMR). In the simulations and in vitro experiments, our technique minimized metabolite signal fluctuations and maintained reliable apparent exchange rate constants. In addition, the apparent exchange rate constants of the metabolites showed differences between the irradiation and control groups after using our method. Comparing the in vitro results obtained using our method and NMR, both solutions showed consistency when uncertainty was considered, demonstrating that our method can accurately measure metabolite signals and show how glycolytic flow changes. The method enhanced the signals of the metabolites and clarified the metabolic phenotyping of tumor cells, which could benefit personalized health care and patient stratification in the future.

## 1. Introduction

Cancer’s primary metabolic pathways require carbohydrates [1]. Since Otto Warburg postulated that glycolysis is the dominant process in cancer [2], it has been known that most cancer cell types consume copious amounts of glucose and lactate [3]. Pyruvate is metabolized into lactate and alanine in the anaerobic glycolytic process. In contrast, pyruvate can also be converted to carbon dioxide and acetyl-CoA through pyruvate dehydrogenase (PDH) in the tricarboxylic acid (TCA) cycle for other glycolytic processes. The metabolite variation may reflect the tumor phenotype and treatment response [4]. For example, Lactate Dehydrogenase (LDH) activity is five-fold higher in cancer. Thus, this feature could be used to evaluate tumors or tissues in vivo and in vitro activity in metabolic imaging [5,6,7].

In MRI metabolic imaging, carbohydrates can be labeled as ^13^C substrates for the MRI measurements, since ^13^C spectra cover a wider range of chemical changes than ^1^H; however, ^13^C spectroscopy has the drawbacks of a low natural abundance (1.1%) and a small gyromagnetic ratio of about a quarter of the ^1^H value. Dissolution Dynamic Nuclear Polarization (dDNP) has been developed to enhance the ^13^C signal [8,9]. This approach transfers polarization from paramagnetic centers buried in a glassy frozen solution to nearby nuclear spins [10]. Its signal is 10,000-fold stronger [8]. This approach can be used to visualize LDH, PDH, alanine transaminase (ALT), and TCA.

Among the substrates used in dDNP, HP [1-^13^C]Pyruvate is the most commonly studied, because of its central function in cellular metabolism, ease of hyperpolarization, lengthy T1 relaxation period, and fast transportation through the cell membrane and metabolism. Although HP ^13^C pyruvate exhibits strong signals, the signals of the downstream metabolites may be only one-tenth or one percent of its HP signal. The small portion of the signal transfer from the substrate raises an issue toward the end of the acquisition window. The apparent exchange rate constants of the metabolites are considered as surrogates reflecting metabolite activities such as LDH and PDH. These values with low signal-to-noise ratio (SNR) may introduce discrepancies when interpreting tumor activity and lead to incorrect conclusions. Numerous papers have proposed denoising algorithms to tackle low-SNR issues and efficiently reduced the noise in the dynamic signal. Maximum entropy signal processing has also been used to suppress noise in the ^1^H and ^13^C spectra [11]. Several groups have applied the concept of wavelet analysis in different fields for the denoising algorithm. Based on the discrete wavelet transform (DWT), various wavelet denoising methods such as wavelet shrinkage [12,13,14], wavelet coefficient modeling [15,16], and wavelet transform modulus maxima (WTMM) [17,18] have been developed and shown to be more effective than filtering methods. Recently, one improved Wavelet Shrinkage Denoising algorithm selected the threshold to filter noise by utilizing the approximation and detail coefficients of electron spin resonance (ESR) [19]. Another method utilized Spectral Improvement by Fourier Thresholding (SIFT) to denoise dynamic MRS [20,21]. In addition, Singular Value Decomposition (SVD)-based low-rank denoising methods have been implemented with dynamic spectra [22]. Here, we have proposed a different approach that improves the signal equally well. This method primarily uses the strong pyruvate dynamic signal and the metabolite variations in the kinetic model to estimate the true metabolite signals. This method was validated and tested by simulation and in vitro studies.

## 2. Materials and Methods

### 2.1. Signal Estimation and Correction

Technically, the MR signal of an object is mainly affected by the MR acquisition parameters and background noise. The MR signal of metabolites is also affected by the apparent exchange rate constants of the substrates to the metabolites. As a result, the metabolite signal can be calculated using MR theory [23] and a kinetic model [24]. Typically, the MR metabolite received/observed signal, $S_{m}^{\prime }$, is the combination of the true metabolite signal, *S_m_*, and background noise, *σ_bkg_*.

$S_{m}^{\prime }=S_{m}+\sigma_{bkg}$, where $S_{m}^{\prime }$ is the received or observed metabolite signal, *S_m_* is the true metabolite signal, and $\sigma_{bkg}$ is the estimated background noise, which can be a positive or negative value. The background noise in the frequency domain at each acquisition was estimated from the ratio of the standard deviation of the tail points of Free Induction Decay (FID) signals and the maximum FID signal in the time domain, and was scaled by the maximum FID signal in the frequency space. The real part of the FID signals in the tail points was averaged and thus considered as the averaged background noise. Whether the mean background noise values in all acquisitions were positive or negative was evaluated, indicating whether the metabolite signals were to have background noise added or subtracted.

Our method began with an estimate of the background noise and evaluation of the sign of the averaged background noise. A gaussian filter, which mainly smoothed the data points, was then used to filter the metabolite dynamic signals, except for pyruvate and lactate, due to the high signal-to-noise (SNR) ratio.

The apparent exchange rate constants of lactate, alanine, bicarbonate, and aspartate were estimated twice by fitting the solutions of Equations (1) and (2) to these metabolite signals simultaneously. In the first estimation, these rate constants were calculated by fitting solutions of Equations (1) and (2) with “raw” metabolite signals, which were defined as the signals as they were before applying our method of rectification. These apparent exchange rate constants were influenced by the metabolite signals, signal variations, relaxation rates, and background noise. These exchange rate constants were treated as the reference values in Equation (2) for metabolite signal correction.

(1) $$\frac{dP\left(t\right)}{dt}=-\underset{i}{\sum }k_{P,M_{i}}P\left(t\right)-\rho_{1}P\left(t\right)$$

(2) $$\frac{dM_{i}\left(t\right)}{dt}=k_{P,M_{i}}P\left(t\right)-\rho_{2}M_{i}\left(t\right)$$

*P*(*t*) is the hyperpolarized signal of the substrate and *M_i_*(*t*) is the downstream metabolite signal, where “*i*” represents each metabolite such as alanine or bicarbonate. The relaxation rates of *ρ*_1_ and *ρ*_2_ both in simulations and in vitro experiments were fixed.

The true signal, $S_{m},$ will most likely be underestimated if the product of the metabolite signal, $S_{m}^{\prime },$ and the averaged background noise is negative. Otherwise, the true signal would likely be overestimated. Both an underestimated and overestimated signal would cause discrepancies in the apparent exchange rate constants in the kinetic model. Thus, we compared the slope of the dynamic “raw” signal with the kinetic model in each metabolite. If the slopes of the “raw” metabolite signal in the underestimated case were larger than 40% of the metabolite variation calculated by the kinetic model, the “raw” signals at these time points were added to half of the background noise. If the slopes of the “raw” metabolite signal in the overestimated case were less than 40% of the metabolite variation calculated by the kinetic model, the “raw” signals at these time points had half of the background noise subtracted from them. The slope was defined as the difference between the metabolite signal at a certain point in time and the subsequent signal, all divided by the time period: Slope(*t*) = (*S*(*t* + 2) − *S*(*t*))/2 (unit: s^−1^), where the number “2” represents the repetition time. The metabolite signal in the underestimated case was added to half of the background noise for each iteration, but in the overestimated case, half of the background noise was subtracted from it for each iteration. The signal correction iteration time was determined by the SNR of the metabolites. The signals of these metabolites were updated and the new apparent exchange rate constants were calculated in the solutions of Equations (1) and (2) with the processed signals. Typically, these rate constants of the processed signals approach *p*-values less than 0.01 after two rounds.

### 2.2. MR Dynamic Signal Simulations

**Figure 1:**
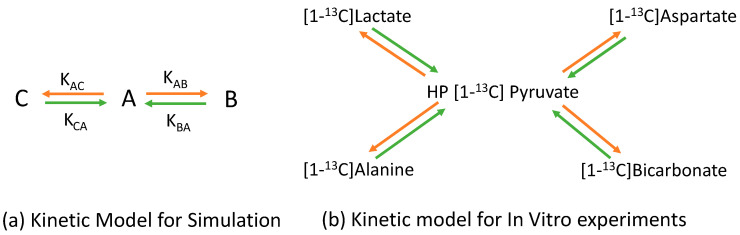
Schematic presentation of the metabolite kinetic model for the simulation and in vitro studies. The orange arrows represent a substrate converting to metabolites [1,32]. The green arrows represent metabolites converting to the substrate. (**a**) “A” is the substrate and “B” and “C” are metabolites transferred from the “A” substrate. (**b**) HP [1-^13^C]Pyruvate is the substrate. Other metabolites are transferred from the HP [1-^13^C]Pyruvate.

FID signals for three ^13^C metabolites (A, B, and C) at 3T were simulated by [23]. The signal evolutions of the three metabolites were based on the two-site exchange kinetic model shown in Figure 1a. The phase of the FID signal was added to a constant phase, 0.95 radian. The phase correction of the whole spectrum after the Fourier Transfer process was 1.1 radian. We added the large and small Rician noise into the FID to, respectively, simulate the high and low SNR of the spectra. The simulation parameters were as follows: there were 4096 sampling points and the total sampling time was 2 s; a zero-order phase of 0.95 radian was added; the T1 value of the three metabolites was 40 s; the forward apparent exchange rate constants of A to B and A to C in the simulations, the orange arrows in Figure 1a, were 1.0 × 10^−2^ s^−1^ and 6.0 × 10^−3^ s^−1^, respectively; the backward apparent exchange rate constants of B to A, and C to A, the green arrows in Figure 1a, were 1.0 × 10^−3^ s^−1^; the chemical shifts of A, B, and C were 28.1 ppm, 46.7 ppm, and 34.0 ppm, respectively; and there were 42 time points, 83 s.

### 2.3. In Vitro Study: Cell Preparation and Irradiation

Human FaDu squamous carcinoma cells were purchased from the American Type Culture Collection (Rockville, MD, USA) and maintained in MEM, RPMI-1640, and DMEM medium (Gibco, Thermo Fisher Scientific, Waltham, MA, USA). FaDu is a human cell line established from a hypopharyngeal squamous cell carcinoma [25]. It has moderate radiosensitivity [26,27,28] and shows alternations in its signal pathways after irradiation [29]. All culture mediums contained 10% fetal bovine serum (Gibco) and 1% penicillin-streptomycin (Gibco). The cells were incubated at 37 °C in a humidified 5% CO_2_ and 95% air atmosphere. They were trypsinized after PBS washing, and the cell numbers and viability were determined using a LUNA-FLTM dual fluorescence cell counter (Logos Biosystems, Gyeonggi-do, Korea). The cell numbers, including the irradiated and control groups, ranged from 3.5 × 10^7^ to 4.4 × 10^7^, maintaining a cell viability of approximately 80%. The cells were then centrifugated and resuspended in a 9 mL mixed medium (1 mL of used medium and 8 mL of fresh medium). The cells were then separated into two groups using 6-MV X rays from a linear accelerator at a dose rate of 6 Gy/min: those that received 15 Gy radiation and those that did not. The cells were subsequently injected with 80 mM hyperpolarized [1-^13^C]Pyruvate (made by GE SPINlab, GE Healthcare, Chicago, IL, USA) for 1 mL for ^13^C DNP-MRI (GE MR750w) scanning using a mouse coil (RAPID Biomedical, Rimpar, Bavaria, Germany). The cells were centrifuged for 2500 rpm to separate the media from the cell pellet after one hour of ^13^C DNP-MRI. We did not perform any cell extraction. Each cell line’s supernatants were diluted to 0.5 mL, half-mixed with a ^13^C NMR buffer (20 mM TSP/D2O), transferred to 5 mm NMR tubes (Bruker BioSpin, Rheinstetten, Germany), and the ^13^C and ^1^H spectra were then recorded using the Bruker 600 MHz NMR spectrometer.

### 2.4. [1-^13^C]Pyruvate Hyperpolarization and In Vitro Experiments

Research-grade fluid paths (RFP; GE Healthcare, Chicago, IL, USA) were filled with 35 mg of [1-^13^C]pyruvic acid doped with a 15 mM electron paramagnetic agent (trityl radical AH111501; GE Healthcare) and 14 g of water containing a 0.1 g/L ethylenediaminetetraacetic acid (EDTA) dissolution medium. Samples were polarized using a clinical hyperpolarizer (SPINlab; GE Healthcare) at a temperature of 0.8 K and a magnetic field of 5 T for an average of 180 min. Following rapid dissolution, the pyruvic acid solution was neutralized and diluted with a TRIS buffered NaOH solution to obtain approximately 5 mL of [1-^13^C]Pyruvate solution at neutral pH. Next, 1 mL of the fluid, containing approximately 75 mM of hyperpolarized [1-^13^C]Pyruvate, was immediately added to 9 mL of the cell suspension in a syringe, resulting in a final pyruvate concentration of 7.0–7.5 mM. The time interval between the dissolution of the hyperpolarized [1-^13^C]Pyruvate and the start of the ^13^C-MRI acquisition ranged from 62 to 77 s (median = 65 s). The temperature of the samples was regulated at approximately 37.0 °C during MR imaging. The pH of the samples ranged from 5.9 to 6.4. The experiments were repeated for the FaDu cancer cells (*n* = 3) in the irradiated and control groups. The cell temperature in one of three FaDu studies was room temperature. In addition, the cells in this FaDu study did not have acetic acid added to the fresh medium before mixing [30].

### 2.5. Data Analysis

#### 2.5.1. In Vitro Analysis

The data were acquired using the pulse-and-acquire sequence. The original spectroscopy data were reconstructed, apodized, phase-corrected, and background subtracted using a “home-made” MATLAB script and some supported functions in the MNS package. The dynamic metabolite signals of [1-^13^C]Pyruvate, [1-^13^C]Lactate, [1-^13^C]Alanine, [1-^13^C]Bicarbonate, [1-^13^C]Aspartate, and [1-^13^C]Malate were extracted in the spectrum by calculating the individual chemical shift, bandwidth, and sampling points. The data after this generic post-processing were called the “raw” data (see Figure S1). The “processed” data were what resulted from the procedure described in Section 2.1.

Equations (1) and (2) represent the kinetic model for the metabolite signals, which were either raw or processed data. The backward terms of the kinetic model, the green arrows in Figure 1b, were ignored due to the strong hyperpolarized substrate signal. The apparent rate constants, K_P,Mi_, were calculated by fitting the dynamic metabolite signals into the numerical solutions of Equations (1) and (2) simultaneously. Based on our past measurements [30] and the values reported in the literature [31], the T1 value of Pyruvate and lactate was assumed to be 45.4 s and the T1 of the other metabolites was assumed to be 25 s.

#### 2.5.2. Simulations

The spectrum contained three metabolite signals: A, B, and C. The dynamic signal of each metabolite was extracted by calculating the individual chemical shift and sampling points. We used the spectra of high SNR as the reference and compared the results of the reference, raw, and processed signals using the following formula, *h*(*t*):

(3) $$h{\left(t\right)}_{a}=\sqrt{\underset{t}{\sum }{\left(f{\left(t\right)}_{a}-g{\left(t\right)}_{a}\right)}^{2}}$$

where *f*(*t*) represents one of the metabolite dynamic signals in either processed or original raw form, *g*(*t*) represents the signal reference, *h*(*t*) reflects the deviation of the processed or raw spectrum line from the reference line, and “a” represents each metabolite. A smaller value of *h*(*t*) means the signal lines are closer to the reference lines. The extreme case, *f*(*t*) = *g*(*t*) causing *h*(*t*) = 0, means that the metabolite signals match the references perfectly. In addition, the apparent exchange rate constants of metabolite B and C were also calculated from the solutions of Equations (1) and (2) with either raw or processed signals.

## 3. Results

### 3.1. Simulation

**Figure 2:**
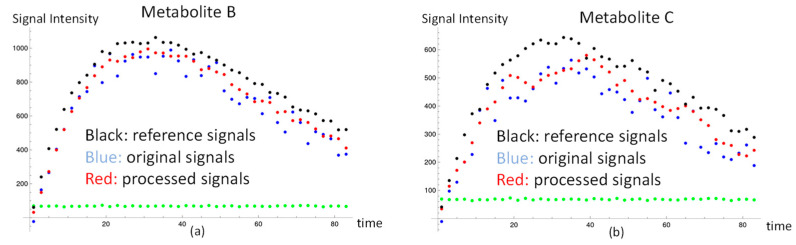
Dynamic metabolite signals and background noise in the simulations. The metabolite signal simulations of high-SNR and low-SNR are shown in (**a**,**b**). The black dots represent the high-SNR simulation. The blue dots and the green dots represent, respectively, the signal and background noise in the low-SNR simulation. The red dots represent the processed signals after using our method. The background noise is the same order of magnitude in both metabolites. (**a**) The reference, raw, and processed signals of metabolite B and the background noise. (**b**) The reference, raw, and processed signals of metabolite C and the background noise.

Here, we present the results of the large and small noise cases using our method. The black dots of metabolite signals B and C in Figure 2a,b were treated as the reference, and represent the small noise case. The end of the SNR of metabolite C in the reference, the black dots, was about 20. The blue dots in Figure 2a,b were the original data of metabolite signals B and C, and represent the large noise case. The red dots in Figure 2a,b were metabolite processed signals B and C after signal correction using our method. The signal differences of metabolite B and C in Equation (3) after applying for our method were decreased by 44%, and 34%, respectively. It is clear that the signal corrections of metabolite B and C were improved by our method. Regarding the kinetic model evaluation, the apparent exchange rate constants of metabolite B and metabolite C, respectively, (9.52 ± 0.01) × 10^−3^ s^−1^ and (5.64 ± 0.06) × 10^−3^ s^−1^, agreed with the expected values, with a six percent difference in the case of small noise. In addition, the apparent exchange rate constants of metabolite B and metabolite C using our method without gaussian filtering of the signals, respectively, (9.52 ± 0.01) × 10^−3^ s^−1^ and (5.52 ± 0.07) × 10^−3^ s^−1^, also agreed with the expected values. These results indicate that our simplified kinetic model, ignoring the backward apparent exchange rate constants, is a feasible tool to accurately calculate the apparent exchange rate constants for the downstream metabolites from the HP substrate. In addition, using the gaussian filter with our method would not affect the apparent exchange rate constants. From a comparison of the metabolite apparent exchange rate constant using the raw data and processed data in the case of large noise, the set-up apparent exchange rate constant of metabolite B was seen to be higher than that of metabolite C by about one order of magnitude. Thus, the signal of metabolite B was large enough that the estimated apparent exchange rate constants K_AB_ were stable before and after signal correction using our method. In other words, our method would not affect the data in the high-SNR case; however, the set-up apparent exchange rate constant of metabolite C was low, and so the signal of metabolite C from substrate A was easily affected by the noise. The SNR of metabolite C shown in the blue dots toward the end time point was close to three. The estimated apparent exchange rate constant, K_AC_, of metabolite C was improved and the result agreed with the expected value with a 12 percent difference. The resulting K_AC_ after applying our method was also improved, as suggested by the data in Table 1. We also investigated the effect of erroneous fixed parameters in the model to the apparent exchange rate constant determination in Table S1. The estimated apparent exchange rate constant in the simulated signals would be modified by the kinetic model with the incorrect fixed T1 value. To make up for the inaccurate greater T1 value, the estimated apparent exchange rate constant can be lower than the predicted value with the bigger T1 in the model.

### 3.2. In Vitro Study

The visible signals of the downstream metabolites in the HP ^13^C pyruvate in the in vitro studies were lactate, alanine, bicarbonate, aspartate, and malate. Although the malate was visible in the irradiated groups, its signal was too weak to have a reasonable apparent exchange rate constant. Thus, our kinetic model did not include it.

#### 3.2.1. Before and after In Vitro Signal Correction

**Figure 3:**
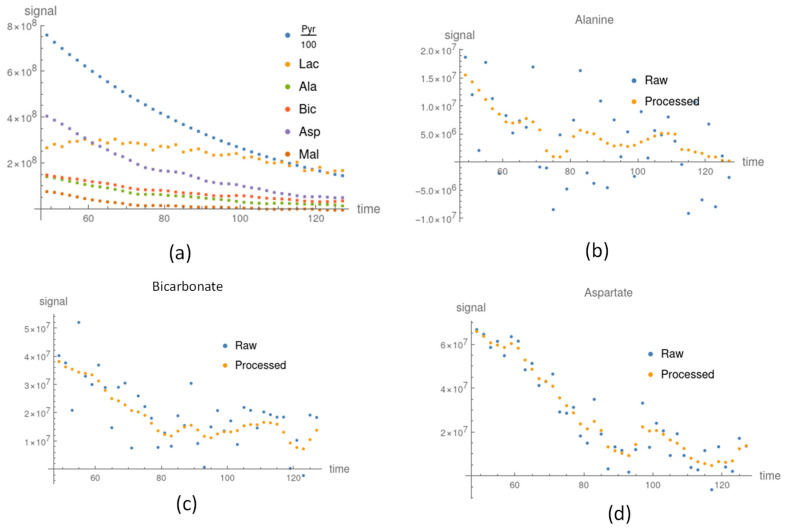
The processed signal after applying our proposed method. (**a**) Pyruvate and downstream metabolite processed signals in the irradiation group. In the control group, the raw signal was compared to the processed signal for (**b**) Alanine, (**c**) Bicarbonate, and (**d**) Aspartate.

After applying our method, the processed signals of Alanine, Bicarbonate, and Aspartate were improved and the signal variations were reduced, as shown in Figure 3b–d. We compared the metabolite apparent exchange rate constant and ratio of lactate and bicarbonate for the processed data, raw data, and NMR measurements in Table 2. The apparent exchange rate constants of pyruvate to lactate between the processed and raw data were stable in the irradiated and control groups. The amount of lactate in the irradiated group was larger than that in the control group. Prior to using our method (see Table 2), the apparent exchange rate constant of Bicarbonate in the irradiation group was lower than that in the control group; however, after using our method, the Bicarbonate apparent exchange rate constant in the irradiated group became marginally higher than that in the control groups. These findings were congruent with those obtained from the NMR measurements shown in Table 2. The rest of the apparent exchange rate results using the processed and raw signal are listed in Table S2 and Table S3, respectively.

**Figure 4:**
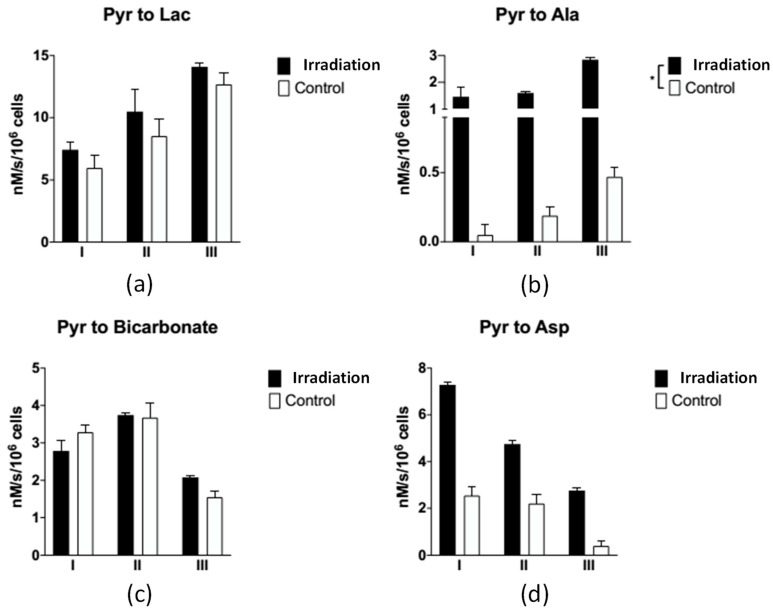
The apparent exchange rate constants of each metabolite between the irradiated and control groups for three measurements. These numbers are listed in Table S2. (**a**) Pyruvate to lactate; (**b**) pyruvate to alanine (“*” represents *p* < 0.05); (**c**) pyruvate to bicarbonate; (**d**) pyruvate to aspartate.

The signals of Alanine and Aspartate in the control groups were weak, as shown in Figure 3b,d, and so the apparent exchange rate constants were not stable and their uncertainties were large. After applying our method, the uncertainties were reduced and the apparent exchange rate constants were consistently lower than those in the irradiated groups (see Table S2 and Figure 4). The signals of lactate were large compared to the other downstream metabolites shown in Figure 3a. The apparent exchange rate constants of pyruvate to lactate were consistent for the processed and raw data. The apparent exchange rate constants of pyruvate to lactate in the irradiated groups were consistently larger than those in the control group.

#### 3.2.2. A Metabolite Signal Comparison between Irradiated and Control Groups

**Figure 5:**
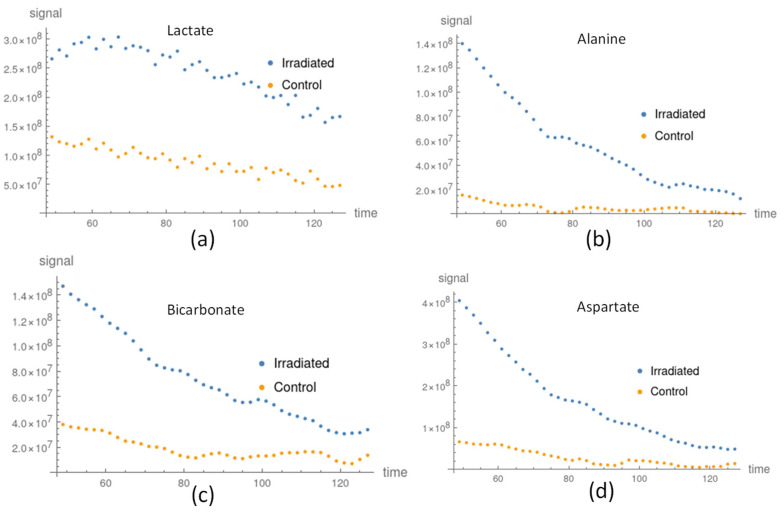
The in vitro metabolite processed signal comparison between the irradiated and control groups: (**a**) lactate signal in the irradiated (blue) and control (orange) groups; (**b**) alanine signal in the irradiated (blue) and control (orange) groups; (**c**) bicarbonate signal in the irradiated (blue dots) and control (orange) groups; and (**d**) aspartate signal in the irradiated (blue dots) and con-trol (orange) groups.

In general, the metabolite signal response of the FaDu cells, such as lactate, Alanine, and Aspartate, in the irradiated group was larger than that of the control groups, as shown in Figure 5. In addition, the apparent exchange rate constants of lactate, alanine, and aspartate were larger in the irradiated groups compared to the control groups, as shown in Figure 4. Considering the uncertainty of the apparent exchange rate constants, those of pyruvate to alanine and aspartate in the irradiated groups were larger than those in the control groups. Although the apparent exchange rate constants of pyruvate to lactate in the irradiated group were larger than those of the control groups, these values did not vary significantly given consideration of their uncertainty in both groups. Two-thirds of the findings in the comparison of the apparent exchange rate constants and NMR measurements in lactate and bicarbonate to pyruvate agree with each other, as shown in Table 3. Both answers are consistent when the uncertainty factor is taken into account. The NMR in vitro measurements are listed in Table S4.

## 4. Discussion

Our method has demonstrated a reduction in signal fluctuations in low-SNR cases by utilizing the features of the kinetic model and background noise. The apparent exchange rate constants were fitted using the processed signals, and the results of the metabolites were consistent with the expected values obtained from the simulations and in vitro studies. The advantage of this method is that it estimates signals directly rather than extracting signals by estimating the background noise through the implementation of a denoised algorithm such as the wavelet or SVD method. When the signal variation of the downstream metabolites is similar to the fluctuation of the background noise, the estimated signal will have a large discrepancy. From empirical observation of the simulations and in vitro data, the obtained kinetic rate of an individual metabolite was not a reasonable value for the majority of SNRs below five. In other words, the SNR limitation for using this method to estimate metabolite signals is a five-to-one ratio. Prior to using this method, the metabolic pathway needs to be known in advance to properly apply the kinetic model, and it is best to measure generic metabolites with NMR or high-field MRI.

Dittmann et al. reported that nuclear EGFR plays a role in triggering the inhibition of pyruvate dehydrogenase and blocking the tricarboxylic acid cycle, and nEGFR triggers a metabolic switch to lactate production in response to irradiation-identified mRNAs associated with the Warburg effect [29]. In addition, Krysztofiak et al. reported that mitochondrial shutdown occurs at the early stages after irradiation [33]. This indicates that cancer bioenergetic fuel processes primarily go through anaerobic glycolytic pathways.

The data generated by the pulse-and-acquire FID sequence were used to generate the in vitro results. We can think of the overall received metabolite signal at each acquisition as a voxel. ^13^C images from the literature, such as least-squares estimation (IDEAL) spiral chemical shift imaging (CSI), may show lactate or other metabolites in some voxels [20,21]. Based on the T1 assumption of metabolites, the apparent exchange rate constants in Table 2 or Table S2, and the acquisition parameters of the IDEAL spiral CSI sequence [20], we can estimate the concentration of metabolites in the voxel that would be visible in HP ^13^C images. In such hyperpolarized ^13^C acquisition, a visible downstream metabolite in a voxel should have a concentration of roughly 50 nM. In contrast, the typical concentration of the detectable signal in MRI is approximately 1 mM. These numbers, 50 nM vs. 1 mM, also echo the HP ^13^C signal enhancement (>44,000) of the original signal observed in past studies [8]; however, only a few downstream metabolites, such as lactate, alanine, and bicarbonate, can satisfy the visible metabolite conditions [30].

The lactate generation time was measured from the time pyruvate was added to the cells to the time the cells were spun to separate the medium from the cell pellet. The apparent exchange rate constants in Table S2 can also be used to compute the overall time length. These numbers did not match the time for the NMR measurements, which was roughly 3600 s. The inconsistency suggests that the apparent pyruvate-to-lactate exchange rate constants may not have been constant during the study period.

The detection of [1-^13^C]Malate and [1-^13^C]Aspartate reflects either increased gluconeogenesis (e.g., the increased activity of anaplerotic pyruvate carboxylase) or incomplete equilibration with fumarate [34,35]. Although gluconeogenesis is only seen in certain specific cells, such as liver cells, the cumulation of fumarate, a well-known oncometabolite, has been shown to occur in cancer cells. The missing piece is the requisite evidence to show an altered fumarate level or fumarate hydratase activity in head and neck cancer cells, namely, FaDu cells; however, evidence has revealed metabolic reprogramming [36,37] and increased 2-hydroxyglutarate [38] levels in head and neck squamous cell carcinoma. The normalization of the altered metabolism could explain the elevated aspartate and malate levels after irradiation, since fumarate can be converted to malate, oxaloacetate, and then aspartate. The signals of [4-^13^C]Malate and [4-^13^C]Aspartate were detected from HP [1-^13^C]Pyruvate in [32]; however, in our overall FaDu studies, the dynamic signal of [4-^13^C]Malate was in the noise region. On the other hand, the signal of HP [1-^13^C]Pyruvate was strong, its shape was wide, and the chemical shift, 178.5 ppm, of [4-^13^C]Aspartate was close to that of [1-^13^C]Pyruvate, 171.1 ppm. The signal of [4-^13^C]Aspartate was mixed with that of HP [1-^13^C]Pyruvate. As a result, we did not include [4-^13^C]Aspartate in our kinetic model.

## 5. Conclusions

Simulations and in vitro experiments indicated that our method for estimating the signals of downstream metabolites from HP [1-^13^C]Pyruvate showed promise with regard to improving accuracy. It improved the signals of the metabolites and elucidated the metabolic phenotyping of tumor cells, which may have future applications in personalized medicine and patient stratification.

## Figures and Tables

**Table 1:** Apparent exchange rate constant results of the simulation studies.

	K_AB_ (×10^−2^) (s^−1^)	K_AC_ (×10^−3^) (s^−1^)
Raw data	1.00 ± 0.04	5.11 ± 0.23
Processed data	1.05 ± 0.03	5.42 ± 0.18

Data are the mean ± one standard deviation.

**Table 2:** Metabolite conversion comparison between the processed data, raw data, and NMR measurement.

	Processed ^a^		Raw ^a^		NMR ^b^	
	IR.	Con.	IR.	Con.	IR.	Con.
Pyr → Lac	10.5±1.8	8.53±1.36	10.5±1.8	8.49±2.56	0.172	0.166
Pyr → Bic	3.74±0.07	3.68±0.39	3.80±0.60	4.03±0.77	0.017	0.016

IR.: irradiated group; Con.: control group; ^a^: apparent exchange rate constants; ^b^: AUC ratio of pyruvate to lactate or bicarbonate.

**Table 3:** FaDu cell medium NMR Results.

	Experiment I			Experiment II			Experiment III		
	Irradiated	Control	Ratio ^b^	Irradiated	Control	Ratio	Irradiated	Control	Ratio
lac/pyr ^a^	8.00 × 10^−2^	1.60 × 10^−1^	5.00 × 10^−1^	1.72 × 10^−1^	1.66 × 10^−1^	1.04 × 10^0^	3.30 × 10^−1^	2.50 × 10^−1^	1.32 × 10^0^
bic/pyr	3.00 × 10^−2^	4.00 × 10^−2^	7.50 × 10^−1^	1.68 × 10^−2^	1.62 × 10^−2^	1.04 × 10^0^	2.48 × 10^−2^	2.69 × 10^−2^	9.20 × 10^−1^

^a^: AUC Ratio of lactate to pyruvate or bicarbonate; ^b^: Ratio: Irradiated/Control.

## Data Availability

The data that support the findings of this study are available from the corresponding author upon reasonable request.

## Supplementary Materials

The following supporting information can be downloaded at: https://www.mdpi.com/article/10.3390/s22155480/s1, Table S1: Apparent Exchange Rate Constant Results in the simulation studies; Table S2: Metabolite Apparent Exchange Rate Constant Results in In Vitro studies; Table S3: Metabolite Apparent Exchange Rate Constant Results by using “raw” signals in the Kinetic Model; Table S4: In Vitro Experiment parameters and NMR measurements; Figure S1: The ^13^C spectrum in irradiated (a) and control (b) groups. The signals of pyruvate and lactate (orange arrows) were pointed. The rest signals of reported metabolites were extracted in the spectrum by knowing the relative chemical shifts to [1-^13^C]Pyruvate.
