# Supplementary material for: Developing a Method to Estimate the Downstream Metabolite Signals from Hyperpolarized [1-13C]Pyruvate
Source: Sensors (Basel). 2022 Jul 22;22(15):5480. doi: 10.3390/s22155480 (PMC9332172; doi:10.3390/s22155480)
Supplement: Supplementary file 1 [file sensors-22-05480-s001.zip › sensors-1773343-supplementary.pdf]

sensors

MDPI

Supplementary Materials

Developing a Method to Estimate the Downstream Metabolite Signals from Hyperpolarized [1-^{13}C]Pyruvate

Ching-Yi Hsieh [1,2], Cheng-Hsuan Sung [1], Yi-Liang (Eric) Shen [3], Ying-Chieh Lai [4], Kuan-Ying Lu [2,4] and Gigin Lin [2,4,*]

[1] Medical Imaging Research Center, Institute for Radiological Research, Chang Gung University, Taoyuan 333, Taiwan; chsieh2016@mail.cgu.edu.tw (C.-Y.H.); a0927001062@gmail.com (C.-H.S.)
[2] Clinical Metabolomics Core Laboratory, Chang Gung Memorial Hospital at Linkou, Taoyuan 333, Taiwan; fantacy52317@gmail.com
[3] Department of Radiation Oncology and Proton Therapy Center, Chang Gung Memorial Hospital at Linkou, Chang Gung University, Taoyuan 333, Taiwan; patience.tw@gmail.com
[4] Department of Medical Imaging and Intervention, Chang Gung Memorial Hospital at Linkou, Chang Gung University, Taoyuan 333, Taiwan; cappolya@gmail.com
* Correspondence: giginlin@cgmh.org.tw; Tel.: +886-3-3281200 (ext. 2575)

The effect of erroneous fixed parameters in the model to the apparent exchange rate constant determination was investigated in Table S1. We generated metabolite signals, A, B, and C, in two groups by assigning different exchange rate constants. These constants of B and C were 1.0x10^{-2}/s, and 6.0x10^{-3}/s, respectively, in the control group. In the contrary, those constants of B and C were 8.0x10^{-3}/s, and 4.0x10^{-3}/s, respectively, in the irradiated group.

Table S1. Apparent Exchange Rate Constant Results in the simulation studies.

fixed T1	35 s		40 s		45 s	
Group	Irradiated	Control	Irradiated	Control	Irradiated	Control
K_{AB} (×10^{-3})	9.46 ± 0.02	7.88 ± 0.02	9.52 ± 0.02	7.54 ± 0.06	9.56 ± 0.35	7.18 ± 0.24
K_{AC} (×10^{-3})	6.10 ± 0.02	4.10 ± 0.01	5.64 ± 0.07	3.60 ± 0.03	5.22 ± 0.20	3.19 ± 0.11

Data are mean ± standard deviation.

Table S2. Metabolite Apparent Exchange Rate Constant Results In Vitro studies.

	Experiment I		Experiment II		Experiment III	
	Irradiated	Control	Irradiated	Control	Irradiated	Control
K_{lac}	7.43 ± 0.58	5.98 ± 1.00	10.5 ± 1.8	8.53 ± 1.36	14.1 ± 0.3	12.7 ± 0.9
K_{ala}	1.46 ± 0.37	0.05 ± 0.07	1.60 ± 0.05	0.19 ± 0.06	2.85 ± 0.08	0.47 ± 0.07
K_{bic}	2.79 ± 0.26	3.29 ± 0.17	3.74 ± 0.07	3.68 ± 0.39	2.08 ± 0.05	1.55 ± 0.16
K_{asp}	7.28 ± 0.10	2.55 ± 0.35	4.75 ± 0.13	2.21 ± 0.38	2.76 ± 0.11	0.41 ± 0.18

Apparent Excahnge Rate Constant (K_i [Pyr]) nM/s/10^6 cells; "i" represents individual metabolite. Data are mean ± standard deviation.

Table S3. Metabolite Results by using "raw" signals into the Kinetic Model.

	Experiment I		Experiment II		Experiment III	
	Irradiated	Control	Irradiated	Control	Irradiated	Control
K_{lac}	7.43 ± 0.58	5.98 ± 2.25	10.5 ± 1.8	8.49 ± 2.56	14.2 ± 2.2	12.9 ± 1.9
K_{ala}	2.33 ± 0.21	0.80 ± 0.20	1.52 ± 0.37	0.08 ± 0.12	2.71 ± 1.80	0.17 ± 0.13
K_{bic}	2.20 ± 0.18	2.64 ± 0.34	3.80 ± 0.60	4.03 ± 0.77	2.16 ± 1.01	2.13 ± 0.37
K_{asp}	7.77 ± 0.59	3.17 ± 0.84	4.69 ± 1.10	1.21 ± 0.62	2.63 ± 1.65	0.02 ± 0.35

Apparent Exchange Rate Constant ($K_{metabolite}$ [Pyr]) nM/s/10^6 cells;

Table S4. In Vitro Experiment parameters and NMR measurements.

Nucleus	Group	Irradiated	Control	Irradiated	Control	Irradiated	Control
	Cell number	1.85E + 07	1.85E + 07	1.75E + 07	1.75E + 07	2.20E + 07	2.20E + 07
	Cell size (μm)			17.2	17.2	16.2	16.2
	Resuspend volume (mL)	9	9	9	9	9	9
	HP-Pyr volume adding (mL)	1	1	1	1	1	1
				RFP dose NMR			
^{1}H	Pyr (mM)			52.11	54.29	55.15	58.67
	Pyr (AUC)			33.01	34.46	36.11	37.13
^{13}C	Pyr-H (AUC)			2.13	2.20	2.44	2.50
	Pyr-H/Pyr			0.06	0.06	0.07	0.07
				Medium NMR			
	Lac (mM)	1.90	1.87	0.83	0.90	2.82	3.07
^{1}H	Pyr (mM)	3.48	4.58	4.70	4.80	5.36	5.79
	Lac/Pyr	0.55	0.41	0.18	0.19	0.53	0.53
	Lac (AUC)	3.23E + 06	6.62E + 06	0.41	0.41	0.81	0.74
	Pyr (AUC)	4.22E + 07	4.02E + 07	2.38	2.47	2.42	2.97
	Lac/Pyr	7.65E − 02	1.65E − 01	1.71E − 01	1.66E − 01	3.34E − 01	2.48E − 01
^{13}C	Bic (AUC)	1.38E + 06	1.57E + 06	0.04	0.04	0.06	0.08
	Bic/Pyr	3.28E − 02	3.91E − 02	1.67E − 02	1.57E − 02	2.38E − 02	2.68E − 02
	Pyr-H (AUC)	2.27E + 06	2.21E + 06	0.16	0.17	0.17	0.18

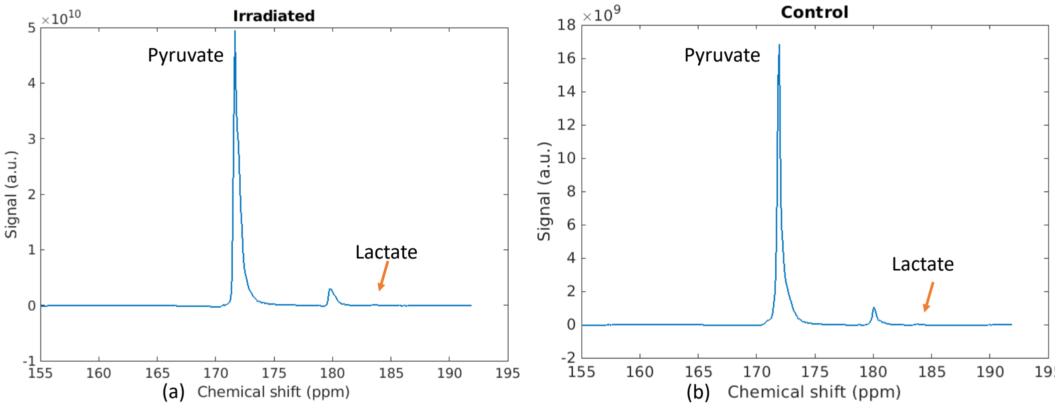

Figure S1. The ^{13}C spectrum in an irradiated (**a**) and a control (**b**) groups. The signals of pyruvate and lactate (orange arrows) were pointed. The rest signals of reported metabolites were extracted in the spectrum by knowing the relative chemical shifts to [1-^{13}C] pyruvate.
